# Return‐to‐sport outcomes after primary and revision anterior cruciate ligament reconstruction using quadriceps tendon autografts in highly active male athletes

**DOI:** 10.1002/jeo2.70718

**Published:** 2026-04-14

**Authors:** Cecilie K. Olsen, Julian A. Feller, Haydn J. Klemm, Kate E. Webster

**Affiliations:** ^1^ Sports Orthopedic Research Center–Copenhagen (SORC‐C), Orthopedic Department Copenhagen University Hospital Amager‐Hvidovre Denmark; ^2^ OrthoSport Victoria Research Unit Richmond Victoria Australia; ^3^ School of Allied Health, Human Services and Sport La Trobe University Bundoora Victoria Australia

**Keywords:** competitive athlete, contact sport, graft selection, high risk, return to performance, return to play

## Abstract

**Purpose:**

To evaluate return‐to‐sport (RTS) outcomes following primary and revision anterior cruciate ligament reconstruction (ACLR) using quadriceps tendon autografts in a highly active male athletic population.

**Methods:**

This retrospective cohort study included 140 highly active male athletes who underwent ACLR with a quadriceps tendon autograft by a single surgeon between 2014 and 2021. Participants were divided into primary (*n* = 93) and revision (*n* = 47) groups. All participants were surveyed regarding RTS outcomes at 2–3 years postoperatively. Survey outcomes included return to some kind of sport, return to preinjury sport (any level), return to preinjury sport at the same or higher level and self‐perceived ability to perform at the pre‐injury level. Descriptive statistics were calculated and presented.

**Results:**

In the primary reconstruction cohort, 98.9% of athletes successfully returned to some sport, 90.3% to their pre‐injury sport, and 73.1% to their pre‐injury sport at the same or higher level. Of those who returned to their pre‐injury sport, 70.2% reported performing at their pre‐injury level. Of those who returned to their pre‐injury sport at the same or higher level, 79.4% reported performing at their pre‐injury level. In the revision cohort, 85.1% of athletes successfully returned to some sport, 57.4% to their pre‐injury sport and 40.4% to their pre‐injury sport at the same or higher level. Of athletes in the revision cohort who returned to their pre‐injury sport, 55.6% reported performing at their pre‐injury level. Of those who returned at the same or higher level, 57.9% reported performing at their pre‐injury level.

**Conclusion:**

Quadriceps tendon autografts yielded high RTS rates in highly active male athletes after primary reconstruction and rates in the revision setting comparable to previous literature. A high proportion of the patients felt they could perform as well as prior to their injury.

**Level of Evidence:**

Level III, retrospective cohort study.

**Abbreviations**
ACLR: anterior cruciate ligament reconstruction
BPTB: bone–patellar tendon–bone
CI: confidence interval
HT: hamstring tendon
IQR: interquartile range
LCL: lateral collateral ligament
LEAT: lateral extra‐articular tenodesis
MCL: medial collateral ligament
PCL: posterior cruciate ligament
QT: quadriceps tendon
RTS: return to sport

## INTRODUCTION

Anterior cruciate ligament reconstruction (ACLR) graft selection has evolved over time. Historically, bone–patellar tendon–bone (BPTB) and hamstring (HT) autografts have been the main grafts used for ACLR. However, concerns regarding harvest site morbidity associated with these grafts have prompted a search for alternative options [8]. In recent years, the quadriceps tendon (QT) autograft has gained popularity as an alternative, even though it remains the primary graft choice for only a minority of surgeons [2]. The QT autograft is increasingly recognized as a valuable option for both primary and revision ACLRs, offering several potential advantages. These include a robust graft that can be harvested with or without a bone block, reduced anterior knee pain, decreased hamstring weakness, improved cosmetic outcomes and a lower risk of graft–tunnel mismatch compared to traditional graft options [7].

When specifically comparing graft types, recent studies suggest that QT grafts yield comparable return to sport (RTS) outcomes to HT grafts in primary ACLR patients [16]. Brinkman et al., in a retrospective cohort of 83 primary ACLR patients with a minimum 5‐year follow‐up, reported higher RTS rates for QT patients (89% return rate) compared to HT patients (80% return rate); however, this difference was not statistically significant [6]. High RTS rates were also shown in a recent study of 656 young active patients who received soft tissue QT grafts, in which 88% of males and 83% of females had returned to their same preinjury sport when surveyed at a minimum of 2 years after surgery [14].

In the revision setting, RTS outcomes are generally lower than those of primary ACLR. A retrospective study of 79 patients found that, regardless of graft type, 89% returned to some level of sport, but only 34% returned to their preinjury level [5]. A systematic review of 606 patients who underwent revision ACLR reported an overall RTS of 77.4% for QT and 65.4% for BPTB grafts. Return to pre‐injury level of play was 58.5% for QT and 50.0% for BPTB, indicating that QT grafts may offer comparable or slightly improved outcomes in the revision setting [3].

In 2014, the treating orthopaedic surgeon (J.A.F.) began using QT grafts more frequently for ACLR, primarily in young, active male patients. Despite increasing adoption of QT autografts, large homogeneous series reporting mid‐term RTS outcomes in exclusively male high‐demand athletes are scarce for both primary and revision ACLR. This study addresses this gap by presenting RTS data in this specific population, offering surgeons a useful benchmark for patients with high RTS demands.

## METHODS

### Study design

Participants provided consent for their clinical data to be included in research studies, and the protocol for this cohort study received ethics approval from the relevant institution (La Trobe University: HEC22221).

### Population

Patients were identified from a practice registry database at a private metropolitan orthopaedic clinic in Melbourne, Australia. This database consisted of patients who had undergone either a primary or revision ACLR between 2014 and 2021.

The study inclusion criteria were male patients who had an ipsilateral QT autograft for their primary or revision ACLR by a single treating orthopaedic surgeon (J.A.F.), who were highly active prior to injury—defined as professional athletes, individuals competing in high‐level competitive sport (non‐professional athletes competing in the highest competitive division of their sport) or those participating in frequent recreational sport (multiple days per week)—who self‐reported their intention of returning to their pre‐injury level of activity following surgery. Exclusion criteria included previous contralateral ACL injury or concomitant injuries to the medial, lateral or posterior cruciate ligaments, and in the case of revision patients, a previous ACL revision procedure.

Only male patients were included to ensure cohort homogeneity. Females represented <15% of QT autograft cases during the study period and were excluded to avoid confounding by sex differences in RTS rates, activity levels, sport types and hormonal/anatomic factors known to influence outcomes. This produced the largest dedicated series in competitive male athletes with QT grafts.

A total of 187 eligible athletes were identified from the database. All were routinely surveyed regarding RTS outcomes between 2–3 years post‐surgery. For some participants, this time point coincided with the COVID‐19 pandemic, during which opportunities to RTS were significantly curtailed, and therefore, a follow‐up survey in 2024 was sent, and the responses from this survey were included in the study. Athletes who had not responded to the initial survey were also re‐sent the survey.

The age, pre‐injury Marx score and pre‐injury sporting level of the study population were compared with two groups of non‐responders: those who completed the initial survey but were affected by COVID‐19 during their rehabilitation and did not respond to the re‐sent survey, and those who did not complete either survey. No significant demographic differences were found between the groups, confirming that the study cohort was representative of the overall population in the database.

### Surgical technique

Graft selection was made by the patient and, in the case of minors, their family, after discussion of graft options with the treating surgeon (J.A.F.). The treating surgeon preferred QT autografts for young, highly active male patients due to the graft's biomechanical strength, low donor‐site morbidity (minimal anterior knee pain, preserved hamstring function) and versatility in primary and revision settings. In the revision ACLR setting, previous graft utilization also influenced the decision‐making.

A lateral extra‐articular tenodesis (LEAT) was used to reduce the risk of graft rupture. The key risk factors for graft rupture that were taken into consideration were young age—with age less than 20 years regarded as a very strong risk factor and age 20–25 regarded as a strong risk factor—a history of contralateral ACL rupture, a family history of ACL rupture and participation at a high level in a pivoting sport such as Australian Rules football, soccer, basketball or netball.

All reconstructions were performed arthroscopically with femoral tunnel drilling via the anteromedial portal. A partial thickness soft tissue QT autograft was used in all cases. The graft was harvested to a standard width of 12 mm. Graft thickness varied between patients, although the target was approximately 5 mm. In cases where the synovium was inadvertently breached during harvest, the tendon defect was repaired using absorbable sutures; otherwise, the partial‐thickness donor site was left open.

Graft length was maximized while avoiding violation of the rectus femoris muscle. The preferred minimum graft length was 7 cm. The proximal end of the graft was secured to a cortical fixation device (EndoButton; Smith & Nephew) using two whipstitches with nonabsorbable suture (Ethibond; Johnson & Johnson, Ethicon). The distal end was similarly whipstitched and fixed in the tibial tunnel with a metal interference screw (Arthrex).

### Rehabilitation protocol

Postoperative rehabilitation followed a standardized protocol overseen by the treating surgeon (J.A.F.) as well as a physiotherapist. The overall progression was symptom‐guided, particularly with regard to pain and joint effusion. Patients were encouraged to progress from home exercises to a gym‐based exercise programme between 6 and 12 weeks. Frequency of review by the rehabilitation provider was variable and dependent upon patient‐specific circumstances, including patient's individual goals, priorities, time constraints and financial situation. Return to jogging was recommended from 12 to 16 weeks and once swelling had resolved. Sports‐specific drills were reintroduced from 16 to 26 weeks. Contact training was introduced gradually over the following 3–4 months. Competitive sport was permitted after confidently completing at least 1 month of unrestricted training—usually between 10 and 12 months postoperatively.

### Outcome measures

Demographic and surgical data were obtained from a prospectively collected institutional database and included age, surgery type (primary or revision ACLR), proximal and distal graft diameters, use of a concomitant LEAT, meniscal status (classified as intact, torn with no repair, torn resected, torn with repair or previously resected for both the medial and lateral meniscus) and presence and location of articular cartilage damage assessed as International Knee Documentation Committee (IKDC) Grade 2 or more (patellofemoral, medial or lateral compartments) [13]. Pre‐injury activity‐related variables were collected through a pre‐surgery survey and included pre‐injury sporting level (categorized as professional athlete, high‐level competitive sport or frequent sport), activity level as measured by the Marx Activity Scale (range 0–16) and the primary sport the patients participated in prior to injury [11].

RTS outcomes were assessed using a post‐operative survey sent via email. Athletes were asked to identify the main sport they hoped to return to and whether they had returned to that sport and at what level (no; yes, at a lower level; yes, at the same level or yes, at a higher level). Additional questions addressed participation in other competitive or recreational sports, specific sports played in organized competition following surgery (including level of participation), the type of recreational sport activities engaged in, a self‐assessment of whether they felt capable of performing at the same level as before their ACL injury, as well as questions relating to any further injuries or complications.

### Statistical methods

All data were analysed using R (Version 4.2.2; R Core Team, 2022). Descriptive statistics were calculated for continuous variables (reported as medians with interquartile ranges [IQRs]) and categorical variables (reported as counts and percentages). Frequency statistics were used to summarize RTS outcomes and patient characteristics. Patients were stratified into age groups based on natural breaks observed in histogram distributions, ensuring clinically meaningful categories that reflected the underlying sample structure. RTS outcomes were reported separately for primary and revision reconstruction groups. No formal statistical comparisons between the primary and revision cohorts were performed, as the study was designed as a descriptive cohort analysis rather than a comparative effectiveness study. The groups differ clinically in important ways (e.g., prior surgery, presence of cartilage damage, revision complexity), and the study was not designed to detect between‐group differences.

## RESULTS

Of the 187 patients who met the inclusion and exclusion criteria, 140 responded to the survey, giving a response rate of 74.9%. The median time to follow up for the primary group and the revision group was 2.81 years (IQR 2.06–4.88) and 4.85 years (IQR 2.62–6.75), respectively.

Table 1 summarizes demographic information for both primary (*n* = 93) and revision (*n* = 47) groups. Both patient groups were highly active with most patients (65% primary cohort; 64% revision cohort) participating in Australian Rules football.

**Table 1:** Demographics.

Total (*n* = 140)	Primary (*n* = 93)	Revision (*n* = 47)
Age (years)		
Median [IQR]	22.3 [18.7, 26.7]	24 [20.4, 29.9]
Range (min, max)	13.8, 47.6	17.4, 52.4
Age (years), *n* (%)		
<20	31 (33.3%)	10 (21.3%)
20–24	27 (29.0%)	17 (36.2%)
25–29	21 (22.6%)	8 (17.0%)
30+	14 (15.1%)	12 (25.5%)
Pre‐Injury Marx Activity Rating Scale		
Median [IQR]	16 [12,16]	13 [12.0, 14.5]
Pre‐injury sporting level, *n* (%)		
Professional athlete	15 (16.1%)	2 (4.3%)
High‐level competitive sports	48 (51.6%)	24 (51.1%)
Frequent sports	30 (32.3%)	21 (44.7%)

Abbreviation: IQR, interquartile range.

Table 2 outlines the surgical characteristics of both groups. Cartilage damage was more common in the revision setting, affecting more than 20% of cases across all compartments.

**Table 2:** Surgical details.

Total (*n* = 140)	Primary (*n* = 93)	Revision (*n* = 47)
Graft diameter proximal (mm)		
Median [IQR]	9 [8.0, 9.5]	9 [8.5, 9.5]
Graft diameter distal (mm)		
Median [IQR]	9.5 [9.0, 9.5]	9.5 [8.8, 9.8]
LEAT, *n* (%)	23 (24.7%)	31 (66.0%)
Medial meniscal injury and treatment, *n* (%)		
Intact	65 (69.9%)	27 (57.4%)
Torn no repair	3 (3.2%)	2 (4.3%)
Torn resected	12 (12.9%)	6 (12.8%)
Torn repair	11 (11.8%)	2 (4.3%)
Previous resection	2 (2.2%)	10 (21.3%)
Lateral meniscal injury and treatment		
Intact	61 (65.6%)	20 (42.6%)
Torn no repair	14 (15.1%)	10 (21.3%)
Torn resected	13 (14.0%)	6 (12.8%)
Torn repair	5 (5.4%)	2 (4.3%)
Previous resection	0 (0.0%)	9 (19.1%)
Cartilage damage (IKDC Grade 2 or more)		
Patellofemoral compartment	3 (3.2%)	11 (23.4%)
Medial compartment	13 (14.0%)	12 (25.5%)
Lateral compartment	4 (4.3%)	11 (23.4%)

Abbreviations: IKDC, International Knee Documentation Committee; IQR, interquartile range; LEAT, lateral extra‐articular tenodesis.

In the primary group, there were eight (8.6%) graft injuries and 11 (11.8%) instances of return to theatre; six for partial medial meniscectomies, three for cyclops lesions and two for arthroscopic debridement.

In the revision group, there were five (10.6%) further graft injuries and seven (14.9%) instances of return to theatre; two for cyclops lesions, two for partial medial meniscectomies, two for partial lateral meniscectomies and one for an arthroscopic debridement.

### RTS following primary ACLR

Table 3 summarizes RTS rates for the primary reconstruction group. All but one of the 93 patients (98.9%) returned to some kind of sport. Returning to the same sport was achieved by 90.3% of athletes. Additionally, 73.1% returned to the same sport at the same or higher level, with age‐specific rates ranging from 64.3% in the 30+ group to 77.8% in the 20–24 group.

**Table 3:** Return to sport rates in the primary reconstruction group.

Primary group															
	Total (*n* = 93)			Age < 19 (*n* = 31)			Age 20–24 (*n* = 27)			Age 25–29 (*n* = 21)			Age 30+ (*n* = 14)		
	*n*/*N*	%	CI	*n*/*N*	%	CI	*n*/*N*	%	CI	*n*/*N*	%	CI	*n*/*N*	%	CI
Return to any sport	92/93	98.9	[94.2–100]	31/31	100	[88.8–100]	26/27	96.3	[81–99.9]	21/21	100	[83.9–100]	14/14	100	[76.8–100]
Return to same sport	84/93	90.3	[82.4–95.5]	31/31	100	[88.8–100]	24/27	88.9	[70.8–97.6]	16/21	76.2	[52.8–91.8]	13/14	92.9	[66.1–99.8]
Return to same sport at same/higher level	68/93	73.1	[62.9–81.8]	24/31	77.4	[58.9–90.4]	21/27	77.8	[57.7–91.4]	14/21	66.7	[43–85.4]	9/14	64.3	[35.1–87.2]

Abbreviation: CI, confidence interval.

Table 4 summarizes self‐reported evaluation of return to performance for the primary reconstruction group. Table 4 shows that 70.2% of athletes who returned to their pre‐injury sport reported performing at their pre‐injury level (95% confidence interval [CI]: 59.3–79.7). Among those who returned at the same or higher level, 79.4% reported performing at their pre‐injury level (95% CI: 66.4–100). Age‐specific RTS rates showed variation.

**Table 4:** Self‐reported evaluation of return to performance for the revision reconstruction group.

Primary group (*N* = 93)															
Felt they could perform at pre‐injury level:	Total (*n* = 93)			Age < 19			Age 20–24			Age 25–29			Age 30+		
	*n*/*N*	%	CI	*n*/*N*	%	CI	*n*/*N*	%	CI	*n*/*N*	%	CI	*n*/*N*	%	CI
Out of those returned to same sport	59/84	70.2	[59.3–79.7]	22/31	71	[52–85.8]	18/24	75	[53.3–90.2]	9/16	56.2	[29.9–80.2]	10/13	76.9	[46.2–95]
Out of those returned to same sport at same/higher level	54/68	79.4	[67.9–88.3]	20/24	83.3	[62.6–95.3]	17/21	81	[58.1–94.6]	8/14	57.1	[28.9–82.3]	9/9	100	[66.4–100]

Abbreviation: CI, confidence interval.

### RTS following revision ACLR

Table 5 summarizes RTS rates for the revision ACLR group. All but 7 of the 47 patients (85.1%) returned to some kind of sport (95% CI: 71.7%–93.8%). Returning to their pre‐injury sport was achieved by 57.4% of athletes, and 40.4% returned to the same sport at the same or higher level (95% CI: 26.4%–55.7%). Rates were highest in the 25–29 group (75%; 95% CI: 34.9–96.8) and lowest in the 30+ group (16.7%; 95% CI: 2.1–48.4).

**Table 5:** Return to sport rates in the revision reconstruction group.

Revision group															
	Total (*n* = 47)			Age < 19 (*n* = 10)			Age 20–24 (*n* = 17)			Age 25–29 (*n* = 8)			Age 30+ (*n* = 12)		
	*n*/*N*	%	CI	*n*/*N*	%	CI	*n*/*N*	%	CI	*n*/*N*	%	CI	*n*/*N*	%	CI
Return to any sport	40/47	85.1	[71.7–93.8]	7/10	70	[34.8–93.3]	15/17	88.2	[63.6–98.5]	8/8	100	[63.1–100]	10/12	83.3	[51.6–97.9]
Return to same sport	27/47	57.4	[42.2–71.7]	6/10	60	[26.2–87.8]	8/17	47.1	[23–72.2]	7/8	87.5	[47.3–99.7]	6/12	50	[21.1–78.9]
Return to same sport at same/higher level	19/47	40.4	[26.4–55.7]	5/10	50	[18.7–81.3]	6/17	35.3	[14.2–61.7]	6/8	75	[34.9–96.8]	2/12	16.7	[2.1–48.4]

Abbreviation: CI, confidence interval.

Table 6 summarizes self‐reported evaluation of return to performance for the revision reconstruction group. Table 6 shows that 55.6% of athletes who returned to their pre‐injury sport felt they could perform at their pre‐injury level (95% CI: 35.3–74.5). Among those returning at the same or higher level, 57.9% reported performing at their pre‐injury level (95% CI: 26.4–55.7). Age‐specific rates varied, with 83.3% in the 20–24 group (95% CI: 35.9–99.6) and 33.3% in the 25–29 group (95% CI: 4.3–77.7).

**Table 6:** Self‐reported evaluation of return to performance for the revision reconstruction group.

Revision group (*N* = 47)															
Felt they could perform at pre‐injury level	Total (*n* = 47)			Age <−19			Age 20–24			Age 25–29			Age 30+		
	*n*/*N*	%	CI	*n*/*N*	%	CI	*n*/*N*	%	CI	*n*/*N*	%	CI	*n*/*N*	%	CI
Out of those returned to same sport	15/27	55.6	[35.3–74.5]	4/6	66.7	[22.3–95.7]	6/8	75	[34.9–96.8]	2/7	28.6	[3.7–71]	3/6	50	[11.8–88.2]
Out of those returned to same sport at same/higher level	11/19	57.9	[33.5–79.7]	3/5	60	[14.7–94.7]	5/6	83.3	[35.9–99.6]	2/6	33.3	[4.3–77.7]	1/2	50	[1.3–98.7]

Abbreviation: CI, confidence interval.

## DISCUSSION

This study investigated RTS outcomes following primary and revision ACLR using QT autografts in a highly active male population. After primary ACLR, 90.2% of athletes returned to their pre‐injury sport, and 73.1% returned at the same or a higher level.

Several studies on QT grafts report promising RTS outcomes, consistent with our findings. Brinkman et al. conducted a retrospective cohort study with a minimum 5‐year follow‐up, reporting RTS rates of 88.9% for QT grafts and 80.0% for hamstring tendon (HT) grafts, with no statistically significant differences [6]. Similarly, Runer et al., in a registry‐based matched cohort study, reported comparable RTS rates for QT (82.1%) and HT (86.7%) grafts [16]. In populations closely resembling ours—active male athletes undergoing ACLR with QT grafts—similar high RTS rates have been observed. For example, Petit et al. reported an 87.7% RTS rate in males aged 14–22 [14], while Balendra et al. reported a 96% RTS rate in professional footballers [4]. These findings collectively support the effectiveness of QT grafts for primary ACLR in physically demanding populations.

The high RTS rates following primary ACLR in our cohort may be partly attributable to patient characteristics. Our group consisted of younger, highly active males, most of whom played Australian rules football—a sport known for its high physical and psychological demands. Younger age and male sex have been associated with greater psychological readiness to RTS, including lower fear of reinjury and higher confidence in physical function [19]. Athletes with strong athletic identity and intrinsic motivation are also more likely to engage consistently in rehabilitation and competitive sport post‐surgery [15].

In the revision setting, RTS outcomes were lower, consistent with prior evidence of reduced RTS rates for revision ACLR compared to primary procedures [1]. In the current study, 85.1% of athletes returned to some form of sport after revision, 57.4% to their pre‐injury sport, but only 40.4% returned at the same or higher level, markedly lower than the primary ACLR group. This decline from overall RTS to returning at the same or higher level supports existing literature. For instance, a systematic review by Ashy et al. reported that, in revision ACLR with QT grafts, 77.4% of athletes returned to any sport and 58.5% to their pre‐injury level [3]. Factors likely contributing to these reduced rates include increased cartilage damage (present in over 20% of revision cases in this study), prior surgical interventions and potential psychological barriers, such as fear of re‐injury [15].

Age‐specific variations in RTS outcomes were evident in both groups. In the primary group, the 20–24 age group exhibited the highest RTS rates at the same or higher level (77.8%), while the 30+ group had the lowest (64.3%). Similarly, in the revision group, the 25–29 age group achieved the highest RTS rate at the same or higher level (75%), while the 30+ group had the lowest (16.7%). These findings are consistent with studies indicating that younger athletes, particularly those in their early 20 s, tend to have higher RTS rates due to greater physical resilience, fewer comorbidities and stronger motivation to return to competitive sport [16, 18]. The lower rates in older athletes may reflect age‐related declines in physical capacity or shifts in priorities away from high‐level sports participation. Indeed, RTS is impacted by many factors, including fear of reinjury, the patient's perception of the recovery of their knee, as well as other life commitments [9, 10].

The self‐perceived ability to perform at pre‐injury levels is a critical outcome to indicate overall satisfaction with surgical and rehabilitative outcomes. Among those in the primary group who returned to the same sport, 70.2% reported that they felt they could perform the same as prior to their injury. For the patients who returned to the same sport at the same or higher level, 79.4% reported that they could perform the same as prior to their injury. These results are slightly higher than those reported in other studies. For instance, Webster et al. reported that 65% of athletes under 20 years of age felt they had returned to their preinjury performance level 3–7 years after surgery [17]. In a later study of patients who played Australian Rules Football (Webster et al.), 61% of athletes who returned to sport reported they had reached their preinjury level of performance [18]. Higher rates in the current study may reflect the elevated baseline fitness and motivation of our population. In the revision group, only 55.6% of athletes who returned to the same sport—and 57.9% of those who returned at the same or higher level—felt they could perform the same as prior to their injury. These findings highlight the additional challenges for a successful RTS faced in revision ACLR, even among highly active athletes. Return to performance is considered an important aspect of a successful RTS [12]. The lower rate of self‐perceived return to performance in patients who had a revision ACLR, in contrast to the primary ACLR group, underscores the importance of addressing not only RTS but return to preinjury performance in both clinical decision‐making and rehabilitation planning.

This study has several strengths, including a consistent surgical technique performed by a single experienced surgeon and mid‐term follow‐up data. While the exceptionally high RTS rates observed here are undoubtedly influenced by the homogeneous cohort of highly motivated, pre‐injury high‐level male athletes, the findings carry meaningful clinical implications. In this demanding population, where RTS is often a primary goal and failure can have substantial psychosocial and professional consequences, QT autografts appear to facilitate excellent mid‐term outcomes. Even in revision cases, where outcomes are typically more guarded, over half returned to their pre‐injury sport and rates aligned with or exceeded literature benchmarks for QT grafts. These data provide surgeons with a reliable reference when discussing graft options with young, active male patients who place high demands on knee function and have concerns about donor‐site morbidity or hamstring weakness associated with traditional autografts.

However, limitations include its retrospective design, reliance on self‐reported outcomes and lack of objective performance metrics. Moreover, the exclusively male and high‐activity cohort limits generalizability to broader ACLR populations. Some participants also underwent rehabilitation during the COVID‐19 pandemic, which may have negatively influenced RTS trajectories, although follow‐up surveys in 2024 mitigated this to some extent. QT autografts were associated with high RTS rates after primary reconstruction and respectable rates in the revision setting, comparable to those previously reported in the literature for QT grafts. These findings support QT as a reliable option in young, active male populations with high expectations and demanding sports, though direct comparison to other grafts is required to determine relative advantages.

## CONCLUSION

QT autografts yielded high RTS rates in highly active male athletes after primary reconstruction and rates comparable to previous literature in the revision setting. Additionally, a high proportion of patients felt they could perform as well as prior to their injury. These results provide surgeons with clinically relevant outcome benchmarks for patient counselling in high‐demand settings.

## AUTHOR CONTRIBUTIONS

All authors contributed to the study conception and design. Material preparation and data collection were performed by Haydn J. Klemm and Cecilie K. Olsen. Analysis and the first draft of the manuscript were written by Cecilie K. Olsen. All authors participated in critically revising the manuscript. All authors read and approved the final manuscript.

## CONFLICT OF INTEREST STATEMENT

The authors declare no conflicts of interest.

## ETHICS STATEMENT

This study was approved by the La Trobe University Human Research Ethics Committee—HEC22221. Patients consented to their medical data being used for research purposes.

## Data Availability

Data are available upon request.
